# Microarrays and RNA-Seq identify molecular mechanisms driving the end of nephron production

**DOI:** 10.1186/1471-213X-11-15

**Published:** 2011-03-12

**Authors:** Eric W Brunskill, Hsiao L Lai, D Curtis Jamison, S Steven Potter, Larry T Patterson

**Affiliations:** 1 Division of Developmental Biology, Cincinnati Children's Hospital Medical Center and the University of Cincinnati School of Medicine, 3333 Burnet Avenue, Cincinnati, Ohio 45229 USA; 2 Division of Nephrology and Hypertension, Cincinnati Children's Hospital Medical Center and the University of Cincinnati School of Medicine, 3333 Burnet Avenue, Cincinnati, Ohio 45229 USA; 3 Division of Biomedical Informatics, Cincinnati Children's Hospital Medical Center and the University of Cincinnati School of Medicine, 3333 Burnet Avenue, Cincinnati, Ohio 45229 USA

## Abstract

**Background:**

The production of nephrons suddenly ends in mice shortly after birth when the remaining cells of the multi-potent progenitor mesenchyme begin to differentiate into nephrons. We exploited this terminal wave of nephron production using both microarrays and RNA-Seq to serially evaluate gene transcript levels in the progenitors. This strategy allowed us to define the changing gene expression states following induction and the onset of differentiation after birth.

**Results:**

Microarray and RNA-Seq studies of the progenitors detected a change in the expression profiles of several classes of genes early after birth. One functional class, a class of genes associated with cellular proliferation, was activated. Analysis of proliferation with a nucleotide analog demonstrated *in vivo *that entry into the S-phase of the cell cycle preceded increases in transcript levels of genetic markers of differentiation. Microarrays and RNA-Seq also detected the onset of expression of markers of differentiation within the population of progenitors prior to detectable *Six2 *repression. Validation by *in situ *hybridization demonstrated that the markers were expressed in a subset of *Six2 *expressing progenitors. Finally, the studies identified a third set of genes that provide indirect evidence of an altered cellular microenvironment of the multi-potential progenitors after birth.

**Conclusions:**

These results demonstrate that *Six2 *expression is not sufficient to suppress activation of genes associated with growth and differentiation of nephrons. They also better define the sequence of events after induction and suggest mechanisms contributing to the rapid end of nephron production after birth in mice.

## Background

In humans, the final number of nephrons that are produced during the formation of the kidney is extremely variable, ranging from 230,000 to 1,800,000 [1]. The number has clinical relevance because a decrease in the number has been associated with hypertension [2]. Although our knowledge about the molecular control of nephron formation has grown substantially in recent years, little is known about the mechanisms controlling the final number.

During renal development, multi-potential progenitors surround the branch tips of the ureteric bud. These self-renewing progenitors are maintained in an undifferentiated state at least in part by Six2 [3], and are induced to differentiate into nephrons by Wnt9b [4]. In mice and humans the lifespan of this population of progenitors is limited providing a means to regulate the final endowment of nephrons. Even though little is known about the mechanism controlling the population's lifespan, it must be able to account for some important differences between humans and mice at the end of nephron production. In humans, for example, branching morphogenesis of the ureteric bud ends early at 22 weeks of gestation [5], whereas in mice it ends within the three days after birth [6]. In humans, nephron production extends twelve weeks beyond the period of ureteric bud branching to 34 weeks gestation, whereas in mice production ends around the same time as branching morphogenesis ends, about three days after birth [6]. Thus, the relationship of the completion of these two processes, branching morphogenesis and nephron production, markedly differ in humans and mice, suggesting the possibility of some small difference in the mechanism controlling the completion of nephron production.

To understand the molecular mechanisms that drive the completion of nephron production in mice, we defined the gene expression programs of the progenitors of nephrons during the first four days after birth. At this time, all remaining progenitors progress from a primarily un-induced to an induced state, and then form renal vesicles. This final wave of production offers a unique opportunity to evaluate the progenitors because of the near synchronous change in their behavior. Our results indicate that induction, defined by altered gene expression, occurs before significant decreases in *Six2*. We also show that proliferation increases prior to detectable increases in transcripts of genes associated with differentiation. Further, we show that expression of some genes, previously defined as markers of later developmental stages, is present in capping mesenchyme cells co-expressing *Six2*. Finally, we observed altered expression levels of genes encoding proteins in the glycolytic pathway, consistent with a change in the microenvironment of the population of progenitors after birth. The microarray results were independently validated and expanded by using a next generation deep sequencing RNA-Seq approach. The resulting profiles better define the order of events and the genes involved after induction of progenitors, as nephron production comes to an end.

## Results

**Figure 1:**
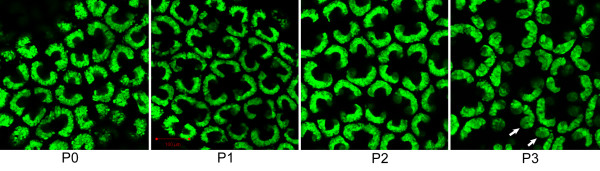
**EGFP expression is limited to the capping mesenchyme and to early stage nephrons after birth, and demonstrates an abrupt change in morphology of the cap**. GFP is expressed in the nephron progenitor mesenchyme of transgenic mice at birth and at lower levels in newly induce nephrons. The GFP signal in the mesenchyme at birth (P0) surrounds the branch tips of the ureteric bud which are unlabeled. The pattern changes very little until post-natal day 3 (P3) when the crescent-shaped mesenchyme begins to be replaced by renal vesicles, ovoid structures with central cavities (arrows). The images are 2 micron thick optical sections taken by a laser scanning microscope on a plane parallel to the tangent of the kidney surface through the cortical nephrogenic zone. The gain was increased at P3 to allow visualization of the reduced levels of GFP expression. The intensities between panels cannot be directly compared.

We captured the green fluorescent protein (GFP)-positive population of cells depicted in Figure 1 from kidneys of Tg(Crym-EGFP)82Gsat/Mmcd mice at birth (P0) and at post-natal days one through four (P1 through P4) by flow cytometry to analyze the levels of gene transcripts. These cells are progenitors that form a cap around the tips of branches of the ureteric bud. Upon induction by the ureteric bud, they begin to differentiate into renal vesicles. In the transgenic mice, they express GFP in a pattern that reproduces *Crym *expression [7]. It has been proposed that the cap can be further subdivided into un-induced and induced mesenchyme based on the expression of Cited1, with the un-induced mesenchyme expressing Cited1 [8]. To better define the population of cells that we would collect by fluorescence activated cell sorting (FACS), we stained the tissues with antibody to Cited1. Both populations, Cited1(+) and Cited1(-), expressed GFP at high levels (See additional file 1a: Optical section of Tg(Crym-EGFP)82Gsat/Mmcd transgenic mouse kidney). The two different cell types were therefore indistinguishable by FACS based on the level of expression of GFP and were captured together during cell-sorting. GFP was also present in renal vesicles at birth and later (See additional file 2: Optical section through transgenic mouse kidney at P0, and Figure 1 P3, respectively), albeit at significantly lower levels. The lower level in differentiating cells made it possible to distinguish them from capping mesenchyme by gating to GFP fluorescence intensity during cell-sorting. GFP was not detected in the stromal mesenchyme between the caps or in the branch tips of the ureteric bud (See additional file 1). The most striking change in character of the cap was seen between P2 and P3 (See additional file 1b) as Cited1 expression turned off leaving only scattered patches of staining at P3. As presented in the discussion, the progenitors on P2 likely represent induced mesenchyme even though they still express Cited1.

The difference in the level of expression of GFP between capping mesenchyme and the renal vesicles allowed us to collect cells of the cap at birth, P1, and P2, and the developing renal vesicles at P3 and P4. We have shown that the multi-potential progenitors, which form the cap and express Cited1, disappear by three days of age in the mouse, and that nephron production ceases at that time [6]. Thus, the cells that are captured at the later times are no longer considered self-renewing progenitors. By gating the FAC sorting to collect only the most highly expressing GFP-positive cells at each time (See additional file 3: Image of the FACS plot of cells collected for RNA measurements:), we could exploit this phase of development to determine the changing gene expression states during induction and early differentiation. The sudden loss of the high-expressing, un-induced mesenchyme after birth and the continued lower-level expression of GFP in the early developing nephron made it possible to collect a series of samples beginning with the un-induced progenitors, then induced progenitors, and finally cells in the early stages of differentiation. This period after birth, which contrasts the embryonic period when the dominant population of GFP-positive cells is un-induced, permits an enriched population of recently induced cells to be isolated.

**Figure 2:**
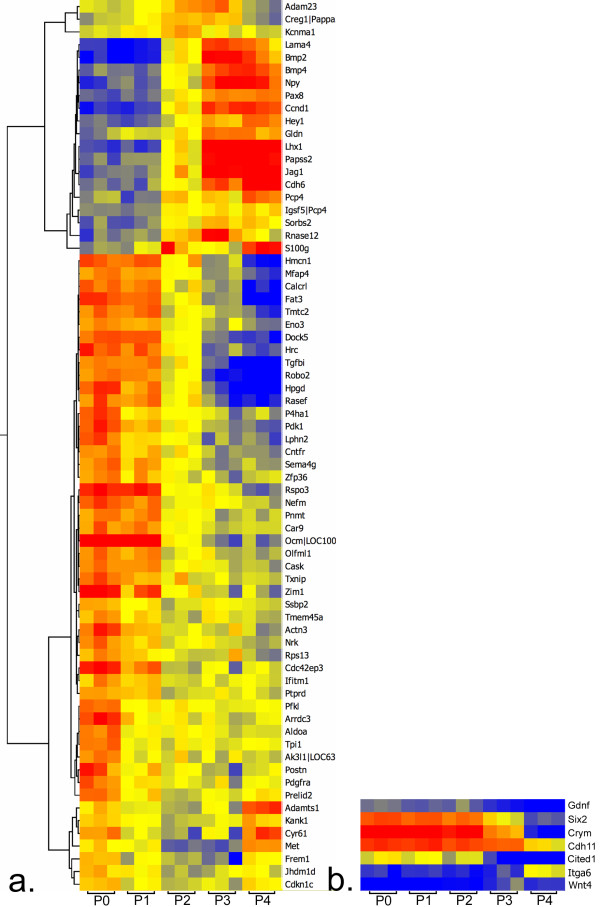
**Heatmap of genes showing significant changes in levels of expression between the progenitor mesenchyme at birth and the mesenchyme at day 2 of life**. Fifteen microarrays, three daily from birth (P0) to post-natal day 4 (P4), were used to define the progression of gene expression in the mouse kidney beginning with the multi-potential progenitor mesenchyme and continuing through induced mesenchyme to early renal vesicles. a) The expression level of twenty genes increased between P0 and P2, whereas the level decreased for fifty genes (blue signifies low, yellow intermediate, and red high levels of expression). The pattern over the entire 4-day period is shown with each column representing the expression levels of a single microarray. Hierarchical clustering divides the genes into groups with similar expression patterns. For example, three genes, *Tpi1*, *Aldoa*, and *Pfkl*, are part of a cluster towards the bottom of the figure. Their levels of expression decreased early, decreasing by P1, and then remained stable through P4. Other clusters of genes with decreasing levels show a continued decrease, or did not demonstrate a decrease until P2. b) For comparison, the heatmap shows the pattern of expression of genes commonly identified with capping mesenchyme. The patterns illustrate changing levels of expression later in the course after birth.

We used a series of microarrays to measure transcript levels in GFP-positive cells from post-natal kidneys. Using ANOVA with Benjamini-Hochberg correction for multiple testing, we identified over 2000 genes with changing levels of expression (p < 0.05) during the four days after birth (See additional file 4: List of genes with changing levels of expression by microarray after birth). This is the most comprehensive list of genes that change in the span between un-induced mesenchyme and renal vesicle. The stepwise sequence in which the genes are activated or inactivated can be easily tracked. For instance, Cited1 expression remained high through P2 before precipitously falling (Figure 2b and See additional file 4), consistent with the expression of Cited1 by immunohistochemistry (See additional file 1b).

Taking advantage of the series of arrays, we next examined a subset of developmental times to detect genes showing very early changes in transcript levels, representing the initial response to either induction or to the extra-uterine environment. By comparing P0 samples with P2 using unpaired t-test, we identified 70 genes (p < 0.05) with at least a 1.5-fold change (Figure 2a). Fifty genes were more highly expressed in the GFP(+) cells at birth than at P2 and included *Tgfbi, Dock5, Robo2*, and *Mfap4*. The expression patterns of these four genes in the cap have historic validation in the embryonic kidney by *in situ *hybridization [7]. Expression was down regulated earlier than a significant down-regulation in expression of *Cited1 *(Figure 2b.), a gene that has been used to mark un-induced cells. The expression of this marker decreased by P2, but it decreased an additional four- to five-fold in the subsequent two days. The genes we identified therefore represent a very early response after birth.

Twenty genes were more highly expressed in the cap at P2 than at P0. They included *Cdh6, Bmp2, Ccnd1, Pax8, Hey1, Lhx1, Npy*, and *Jag1*. They too have historically validated embryonic expression, but in the early stage nephrons, rather than in the mesenchyme. Arrays also identified developmental control genes, such as *Rspo3*, a secreted regulator of beta-catenin signaling [9], and *Fat3 *(see discussion), two genes that have not been studied during renal development. It is interesting to note at P2, when capping mesenchyme is still abundant, that many of the genes showing higher expression compared to P0 are reported in the literature to be associated with more advanced stages of differentiation and not with the mesenchyme. These results could represent either contamination with differentiated cells or low-level expression of vesicle genes in the induced cap prior to vesicle formation.

### *In situ *hybridization expression

**Figure 3:**
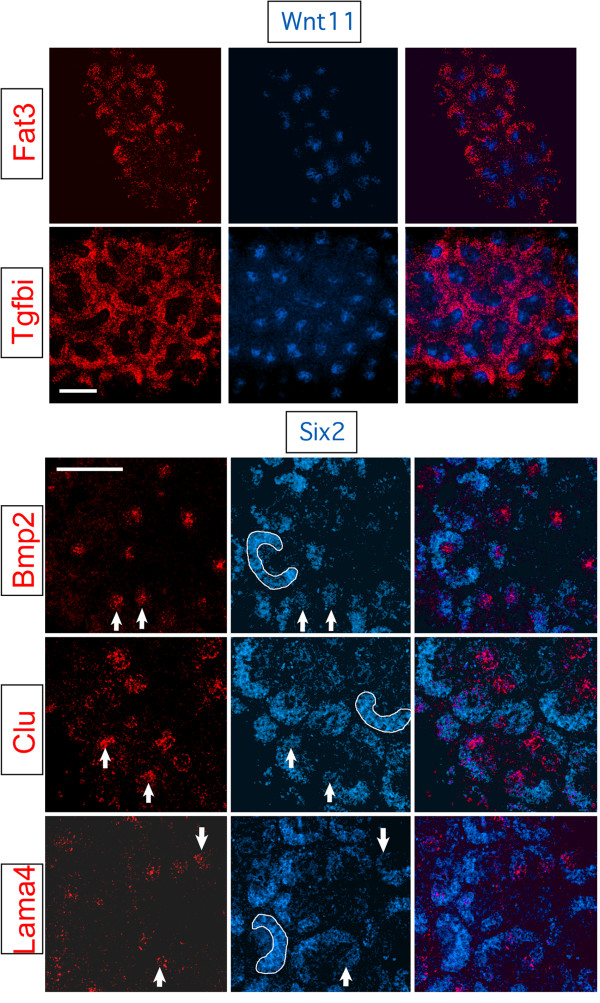
**Dual-label fluorescent whole mount in situ hybridization studies showed the expression patterns of a set of genes which have a changing level of expression after birth**. *Tgfbi *and *Fat3 *were expressed in the nephron progenitor mesenchyme (red signal) surrounding ureteric bud branch tips (blue - *Wnt11 *riboprobe) at P2. *Bmp2*, *Clu*, and *Lama4 *(red) were expressed in the nephron anlage, and were also co-expressed (arrows) with *Six2 *(blue) in capping mesenchyme. Selected regions of capping mesenchyme are outlined with a white line. Bars = 100 microns.

Basing our choices on the microarray results, we selected genes with changing levels of expression to validate their patterns of expression and to address the question of possible contamination. To microscopically localize the expression, we used dual-label fluorescent whole mount *in situ *hybridization. This method provides better localization than other *in situ *protocols because the fluorochrome is covalently bound to tissues at the site of hybridization. It also permits co-localization of expression because confocal imaging can determine expression of genes in thin optical sections. As expected, the expression signals of those genes with higher levels at P0 than P2 by microarray were detected in the cap and not in developing nephrons (*Fat3 *and *Tgfbi*, Figure 3). Some genes (*Bmp2, Clu*, and *Lama4*) having higher expression at P2 rather than P0 were also expressed in the mesenchyme, but the expression was more restricted. The signal co-localized with a cap marker, *Six2*, only at the ends of the crescent-shaped cap (Figure 3), in the region of the definitively induced Cited1(-) cells. The results of the microarrays were therefore validated and not an artifact of contamination by differentiated cells. Nephron anlage did not underlie these areas, indicating that the observed cap mesenchyme expression was genuine and not the result of spillover from adjacent regions as well. These genes were also highly expressed in developing nephrons as were other genes that were similarly up regulated by P2.

### RNA-Seq

We also used RNA-Seq to independently measure differences in gene expression between progenitors at P0 and P2. Deep sequencing of cDNA libraries lacks the bias related to microarrays, and it can be used to digitally quantify gene expression, even at the exon level, over a wide dynamic range. We aligned the sequences to the mm9 mouse sequence database. This is a subset of Ref-seq, which is an integrated, non-redundant database of linked nucleotide and protein sequences. The total number of single-end sequence reads from P0 and P2 was 3.1 and 3.3 million reads, respectively. Alignment with mitochondrial sequences gave an additional 0.65 and 0.4 million reads, respectively. Reads aligned to 19,168 mouse genes and counts ranged from 1 to almost 150,000. Interestingly, and providing a measure of the quality of the analysis, we found that the percent decrease (37%) in expression of GFP at P2 was similar to the decrease (49%) in expression of *Crym*, the gene controlling GFP expression.

**Figure 4:**
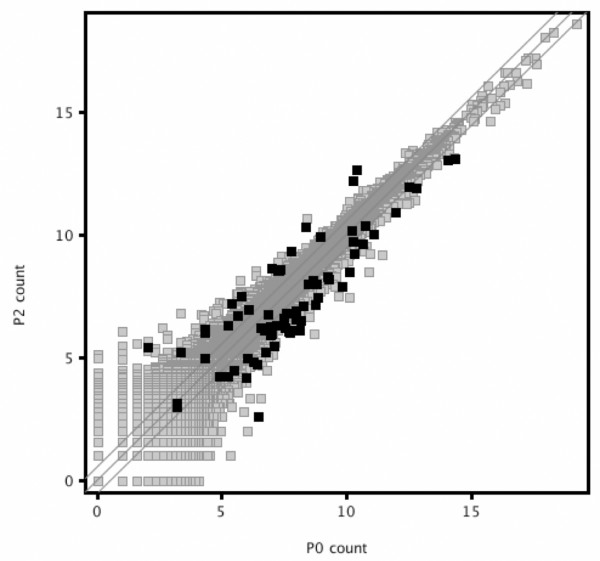
**Scatter plot of the RNA-Seq expression data demonstrates that most of the microarray-defined genes with changing levels of expression (bold) have a concordant change by RNA-Seq**. The plot is on a log_2 _transformed scale.

The RNA-Seq data provided independent high throughput validation of the microarray results. Ninety percent of the genes called at least 1.5 fold differentially expressed between P0 and P2 by microarrays were confirmed by RNA-Seq (Figure 4). Indeed, even with the modest number of sequence reads generated in this study, RNA-Seq found more gene expression differences than microarrays. Analysis with Partek Genomic Suite showed, after Bonferroni correction, that over 300 genes differed significantly in levels of expression at the two times (See additional file 5: List of genes with a change in level of expression by RNA-Seq between P0 and P2). Of these, 64% changed by 1.5-fold or more. In fact, it identified a significant decrease in expression of *Cited1 *by P2 (See Table 1), a decrease that was not discovered by microarray.

**Table 1:** RNA-Seq reads at P0 and P2 for genes expressed during renal development

	P0 (RPKM)	P2 (RPKM)	Significance
**Genes**			
*Cited1*	735	350	P < 10^-93^
*Crym*	5451	2791	P < 10^-200^
*Gdnf*	9	7	NS
*Itga6*	7	8	NS
*Six2*	3	2	NS
*Wnt4*	1	16	NS
*Wt1*	2	2	NS

RPKM - Reads per kilobase per million sequences - normalization to the total number of reads

**Figure 5:**
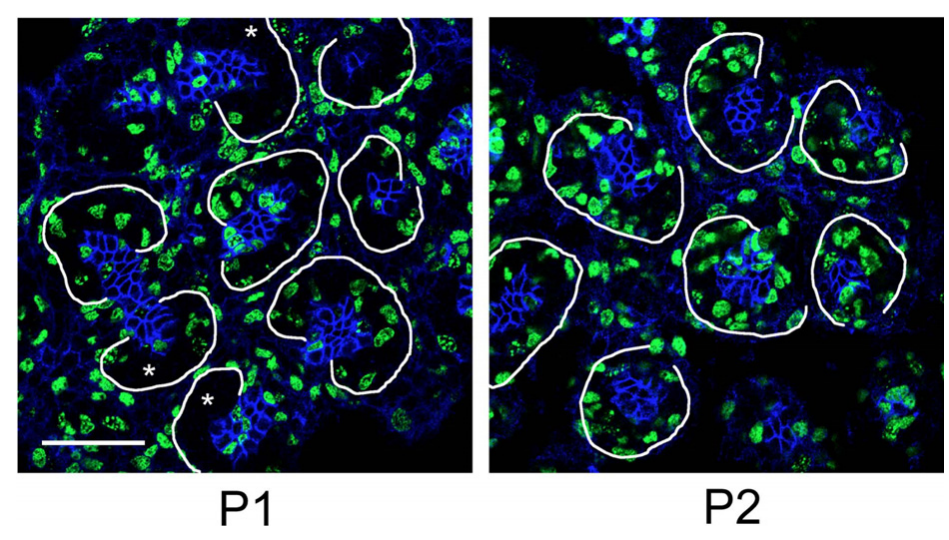
**Cell cycle S-phase labeling by nucleoside analog incorporation with EdU (green) showed a higher rate of cell proliferation in the capping mesenchyme at P2 than at P1**. At P2 the cap surrounding ureteric bud branch tips (blue - E-cadherin) contained many more cells which incorporated the label. The optical sections at P1 and P2 were obtained at the same level relative to the branch tip of the ureteric bud, just superficial to the central lumen in the branch tips of the ureteric bud. Bar = 50 microns.

**Figure 6:**
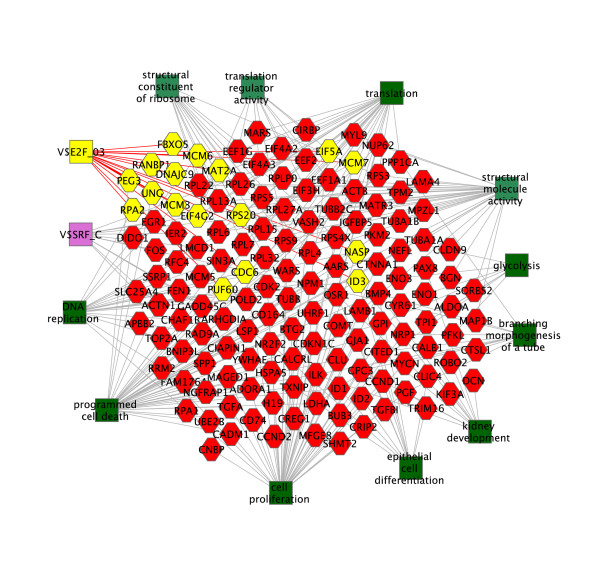
**ToppGene functional analysis of genes changing in expression between P0 and P2**. Green boxes indicate biological processes (dark) and molecular functions (light) with gray lines indicating connections to associated genes (hexagons). V$SRF_C (purple) and V$E2F_03 (yellow) boxes are transcription factor binding sites evolutionarily conserved in the promoter regions of multiple genes. Candidate targets of E2F are shown in yellow. Complete lists of molecular functions, biological processes, and transcriptional factor binding sites and associated genes are in Additional file 6.

We examined the RNA-Seq data to identify classes of genes that were activated early after birth during the induction of progenitors. We identified a strong signature of cell proliferation (*Rrm2, Vrk1, Mycn, Cenpm, Chaf1b, Uhrf1, Fen1, Psat1, Plekhg6, Ccnd1*, and *Usp10*). This suggested that activation of the cell cycle was an early response to induction. We then examined cell proliferation in detail using EdU nucleoside analog incorporation and found a marked difference between P1 and P2 in the distribution of cells entering S-phase (Figure 5). At P1 large patches of capping mesenchyme were devoid of cells incorporating EdU. At the same time incorporation was seen in ureteric bud branch tips, in the stromal mesenchyme, and in some areas of cap, indicating penetration of the label into all structures, and serving as a positive control. A day later, at P2, cells incorporating EdU appeared randomly scattered throughout the capping mesenchyme, occupying the regions where incorporation had been absent. The number of EdU(+) cells increased from 3800 cells/mm^2 ^surface area of the capping mesenchyme at P1 to 5900 cells/mm^2 ^at P2. The increase in number of cells incorporating EdU not only preceded structural changes associated with differentiation, but also seemed to precede the onset of expression of the earliest genes associated with differentiation. At this time the only detectable evidence of activation of genetic markers associated with differentiation appears is at the ends of the crescent-shaped caps (Figure 3). Thus, the studies better define a sequence of events after induction *in vivo*. Consistent with expression of genes associated with proliferation, we also found a significant change in expression of genes with putative transcription factor binding sites for E2F, a regulator of proliferation [10] (Figure 6 and See additional file 6: Functional sets of genes with a change in level by RNA-Seq between P0 and P2). Finally, we found that levels of transcripts for multiple ribosomal proteins changed, suggesting global changes in levels of protein synthesis.

### P1-P4 gene expression differences

**Figure 7:**
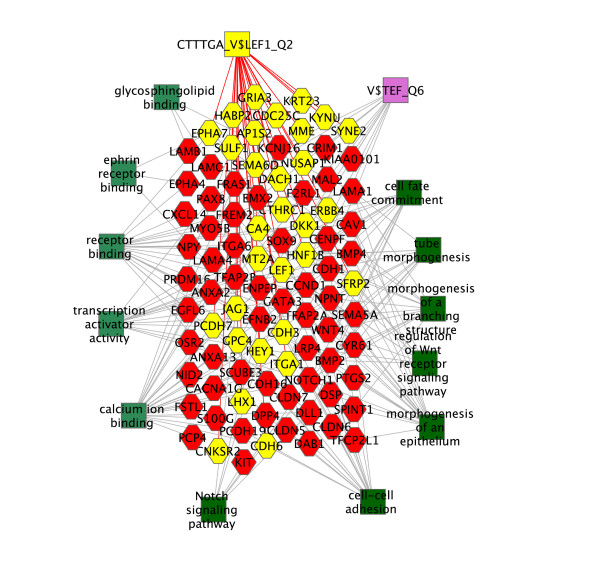
**ToppGene functional analysis of genes elevated in expression between P1 and P4**. Green boxes indicate biological processes (dark) and molecular functions (light) with gray lines indicating connections to associated genes (hexagons). Highly enriched, evolutionarily conserved transcription factor binding sites, V$TEF_6 (purple) and CTTTGA_V$LEF1_Q2 (yellow) are shown, with candidate Wnt (Lef1) target genes (yellow hexagons). Complete lists of biological functions, molecular functions, and transcription factor binding sites are found in Additional file 8.

A comparison of the P1 to P4 gene expression profiles identified changes occurring with further differentiation of the cap mesenchyme into renal vesicles. A fairly stringent screen (paired t-test P < 0.05, including Benjamini-Hochberg correction, and a fold change > 2) found 227 significantly up-regulated and 206 significantly down-regulated genes (See additional file 7: List of genes with changes in transcript levels between P1 and P4). ToppGene analysis of the up-regulated genes identified molecular functions and biological processes including calcium ion binding, ephrin receptor binding, sphingolipid binding, cell adhesion, and epithelium development. In addition, the analysis identified a number of candidate downstream targets of Wnt signaling, as defined by the presence of evolutionarily conserved Lef transcription factor binding sites in proximal promoter regions (Figure 7 and See additional file 8: Functional sets of genes with transcript levels that decrease or increase between P1 and P4).

### Glycolysis in progenitors

Of interest, in our analysis of the microarray data we found that eight genes encoding enzymes in the glycolytic pathway had a significantly reduced level of expression (P < 0.05) at P2 compared to P0. Five of these were down regulated at least 1.5 fold (*Pfkl, Aldoa*, *Tpi1*, *Eno3 *and *Pdk1*). Three of these genes (*Pfkl, Aldoa*, and *Tpi1*) clustered together (Figure 2), indicating similar expression patterns after birth. The pattern of change was evident by 24 hours after birth. The change preceded the increase in *Wnt4 *levels, a marker of renal vesicles, and preceded the decrease in *Cited1 *levels (Figure 2 and See additional file 4). RNA-Seq validated this decrease in level of expression of seven genes (*Gpi1, Pfkl, Aldoa, Tpi1, Eno3, Pkm2*, and *Pdk1*). In contrast, the level of expression of genes encoding enzymes of the Kreb's cycle (*Aco2, Idh2, Ogdh, Scla2, Sdha, Fh1*, and *Mdh1*) remained constant in the interval between P0 and P2 by both microarray and RNA-Seq. We analyzed microarray data from the GUDMAP consortium [11] to identify changes in expression of glycolysis genes during the process of differentiation of cap mesenchyme in the embryo. When we compared the cap mesenchyme expression of E15.5 embryos to renal vesicles of E12.5 embryos, we could not detect similar changes in expression of the glycolysis genes.

## Discussion

The genome-wide evaluation of the multi-potent progenitor cell mesenchyme and early stage nephron transcriptome after birth showed sequential activation or inactivation of many genes. The use of tissue after birth to obtain expression data eliminates potential artifacts introduced during *in vitro *studies of sequential activation after induction. The period after birth allows serial measurements of transcripts early after induction because there is such an abrupt change in the behavior of the progenitors. By P3 all remaining capping mesenchyme has begun conversion into renal vesicles. Because the inductive period and the time to show signs of differentiation after induction each take 12-24 hours (reviewed in Saxen [12]), the final wave of induction to form nephrons had begun by P2. This strongly suggests, therefore, that the Cited1(+) cells at P2, which still constitute most of the GFP(+) capping mesenchyme (See additional file 1), were likely to have been induced and to be different from the capping mesenchyme on P0 and P1 when the bulk of the high GFP expressing cells was un-induced. There is clearly a progression of molecular events post-induction, with the down-regulation of Cited1 corresponding to a more advanced state of induction or possibly a state of commitment.

Our results confirmed differences in expression level between capping mesenchyme and renal vesicle found among a series of markers previously reported by Mugford *et.al*. [8]. Seven of the nine markers, which distinguished between progenitor cell mesenchyme and vesicles by *in situ *(*Cited1, Bbx, Eya1, Osr1, Six2, Dpf3*, and *Meox1*), showed greater than a 2-fold change in level of expression between P0 and P4. The trend was correct for the remaining two markers (*Hoxc5 *and *Brpf1*), but did not reach a 2-fold change in level. Our data extend this list significantly by adding more than 400 genes that change in level between P0, when mesenchyme is abundant, and P4, when GFP primarily marks renal vesicles. This provides a significant resource that can be used when monitoring induction of the mesenchyme.

Discovery of genes that have a spatially restricted pattern of expression during the process of differentiation helps to identify pathways that may be needed for nephron formation. We found that *Fat3 *was expressed in the cap at P0 and was down regulated by P2. The Fat genes encode cadherin-type proteins with cell-cell adhesion properties. With mutation of Fat genes in *Drosophila*, there is overgrowth of tissues [13]. It is interesting in *Drosophila *that Fat is part of a pathway involved in the suppression of wingless, a homolog of a murine gene (*Wnt4*) that is expressed after mesenchymal induction and is required for nephron development [14]. This suggests that the down-regulation of *Fat3 *may be needed before activation of *Wnt4 *in the induced mesenchyme.

We also identified early up-regulated expression of genes in several different pathways and examined them by *in situ *hybridization. One gene, *Bmp2*, was activated early in the induced capping mesenchyme next to one side of the ureteric bud branch tips. The secreted protein encoded by *Bmp2 *is known to regulate both the branching of the ureteric bud tips and the proliferation of their cells [15]. With the localized expression, *Bmp2 *may regulate the regional growth of cells within the tips. Its expression is also of interest because along with *Clu *and *Lama4 *it is activated in the induced cap before morphologic changes are evident. It seems reasonable that capping mesenchymal cells initiating renal vesicle formation would begin expressing genes associated with renal vesicles, although to our knowledge this has not been previously demonstrated.

It is interesting that these induced genes, which are associated with differentiation, are co-expressed with *Six2. Six2 *is necessary to maintain the population of multi-potential progenitors; however, it has not been shown to be sufficient. The co-expression of genes involved in differentiation in a subset of *Six2 *(+) cells suggests that *Six2 *is not sufficient to block transcription of some genes associated with differentiation in the presence of an inductive signal.

We also observed an interesting surge in cell proliferation that preceded the expression of markers of differentiation. The sequence of events suggests the possibility that proliferation promotes the reprogramming of renal progenitors. This type of mechanism has been described during reprogramming of somatic cells into pluri-potent stem cells [16]. An increase by P2 in proliferation, evidence of a change in the behavior of progenitors, is also consistent with induction of the cells by P2.

Also of note, the gene expression profiles of early forming nephrons differed before and after birth. The genes included those encoding enzymes of the glycolytic pathway. The shift in transcription is compatible with a response by the population of progenitors to a change in the microenvironment, such as a post-natal increase in oxygen levels. Prior to birth the kidney is fed by deoxygenated blood, blood with the same oxygen content as that returning to the placenta. In addition, oxygen delivery to the nephrogenic region is further limited because the tissue is relatively avascular. Relative physiologic hypoxia, such as this, is known to cause an increase in transcription of genes encoding enzymes of the glycolytic pathway [17]. Changes in levels of expression of other genes that are regulated in an oxygen-dependent manner, such as *P4ha1*, *Bnip3L*, and *Txnip*, provide further supportive evidence of an increase in oxygenation in the progenitors after birth.

Cellular fates of placental cytotrophoblasts [18], hematopoietic progenitors [19], human neural stem cells [20], bone marrow stromal cells [21], and human embryonic stem cells [22] have been shown to be altered by oxygen. The fate of murine embryonic stem cells also appears to be coupled to metabolism [23]. It seems reasonable, therefore, to speculate that a change in the microenvironment, such as a change in oxygenation within the physiologic range, might also lead to a change in behavior of the multi-potential progenitors in the kidney *in vivo*.

Lastly, the final nephron endowment is clearly a result of regulation of the balance between the rate of progenitor renewal and the rate of differentiation. Simple geometry might play an important role in the balance in both mice and humans. In the early kidney, the capping mesenchyme layer is relatively thick. As branching morphogenesis proceeds, the number of branch tips will expand geometrically, subdividing the capping mesenchyme while also inducing it. Unless renewal of the progenitors similarly expands, the cap around each tip will thin. And, at some point it will no longer be able to promote further branching. After branching ends, if usage of cells to make nephrons exceeds the renewal rate, nephron production will consume the remaining cap mesenchyme. In humans this simple model fits nicely with the completion of nephron production because there is a prolonged period of nephron production without branching. In the mouse, however, there is an abrupt end to both nephron production and branching morphogenesis. The end coincides with birth and with a change in metabolism that is compatible with an increase in oxygenation of the progenitors. Coupled with the known effects of oxygen on cellular fate, the events suggest a possible trigger in mice at birth that shifts the balance between renewal and differentiation of progenitors favoring differentiation. We speculate that the trigger then limits the lifespan of the population of progenitors and causes the production of nephrons in mice to end abruptly.

## Conclusions

In this study we used microarrays, RNA-Seq, *in situ *hybridization, and EdU nucleotide incorporation to examine the synchronous wave of nephron formation that occurs in mouse following birth. The results provide a global definition of the changing gene expression program that drives the transition from un-induced capping mesenchyme, to induced capping mesenchyme, and to renal vesicle. Several genes were found to change in expression before Cited1 was down regulated, suggesting a further molecular subdivision of induced capping mesenchyme. We also observed that cell proliferation preceded differentiation. Further, we observed the expression of renal vesicle associated genes, including *Bmp2*, within the Cited1(-), but Six2(+), region of the capping mesenchyme. This demonstrates that *Six2 *at the normal level of expression alone is not sufficient to block differentiation. Finally, genes essential for glycolysis were down regulated post-birth, compatible with an increase in levels of oxygen within the capping mesenchyme domain. Given the known influence of oxygen on the fate of cells this finding suggests a possible trigger that promotes the final burst of nephron production.

## Methods

### Animals

Tg(Crym-EGFP)82Gsat/Mmcd mice with GFP expression in nephron progenitors were used. To obtain developmentally uniform tissue, mice were housed in standard light-dark cycles, matings were started at midnight, and mice checked for vaginal plugs eight hours later. At noon on day 18 (e18.5) embryos were delivered by Caesarian section, resuscitated, and placed with a foster mother. Each litter of pups was used to obtain kidneys for multiple post-natal samples. Kidneys were dissected at birth (P0) and at 24-hour intervals afterwards (P1-P4). We used CD-1 mice for *in situ *hybridization and nucleotide analog incorporation. The use of experimental animals described in this study complies with the guidelines of the Institutional Animal Care and Use Committee at Cincinnati Children's Hospital.

### Mesenchyme isolation

Two pairs of kidneys were isolated for each sample and three samples isolated for each time. The kidneys were digested in 0.05% Trypsin - EDTA for 15 minutes at 37°C, treated with 2% FBS, and mechanically disaggregated at 4°C. The suspension of cells was filtered through a 70-micron filter and separated using a BD FACS Aria II Cell Sorter to collect GFP (+) cells with the highest fluorescence intensity (See additional file 3: Image of the FACS plot of cells collected for RNA measurements).

### Microarray and data analysis

Fifteen samples, three for each age, were isolated by cell sorting and collected in RLT (Qiagen, CA). 5 ng of total RNA was used with the NuGen WT-Pico, Exon Module, and Fl-Ovation v.2 kits to generate target. 2.5 μg of target was hybridized to Affymetrix Mouse Gene 1.0 ST arrays. The data was deposited in the Gene Expression Omnibus database (GSM429020-GSM429034). Analysis was performed using GeneSpring GX and RMA normalization. COMBAT software was used to correct for batch effect [24]. ToppGene Suite was used to analyze gene lists to identify functional groups of genes [11]. Cytoscape was used to create Figure 6 and 7. To compare post-natal changes in expression of glycolysis genes during differentiation of progenitors to changes in the embryo we analyzed microarray data from the GUDMAP consortium which had been deposited in the Gene Expression Omnibus (GEO, GSE12588 and GSE6290 datasets).

### *In situ *hybridization

A dual-labeled fluorescent whole mount *in situ *hybridization procedure was used with confocal microscopy to validate microarray results. Digoxigenin- or fluorescein-labeled riboprobes were made for each of the test genes (*Clu*, *Fat3*, *Lama4*, *Tgfbi*, *Bmp2*) and for both of the reference genes (*Wnt11 *and *Six2*). Perkin Elmer tyramide signal amplification and the Zeiss LSM 510 microscope were used to detect the hybridization signal. A series of 2-micron thick optical sections were obtained beginning at the surface and extending into the kidney at 5 micron intervals.

### RNA-Seq and analysis

We collected GFP-positive progenitors by fluorescence activated cell sorting, purified the RNA, and synthesized cDNA from 150 ng of pooled RNA from each time, P0 and P2, using the Clontech SMART cDNA synthesis kit. cDNA was then amplified using Stratagene Herculase polymerase during a 24-cycle PCR reaction. The PCR products were purified and treated with the BAL-31 nuclease to remove the SMART cDNA primer end, and sequenced using the Illumina Genome Analyzer II standard 36-cycle paired-end protocol. Sequences were aligned to the *mus musculus *(mm9 sequence database) subset of RefSeq [25] in an unpaired alignment using the ELAND [26]. The alignment could contain only two mismatches of a 32 bp read to be included in the datasets. GeneSpring was used to graphically present the raw RNA-Seq data and Partek Genomic Suites for statistical analysis.

### Cell cycle labeling

We used the Invitrogen Click-iT EdU Alexa Fluor 488 to label cells in S-phase of the cell cycle. Pups were injected with EdU, a nucleotide analog, at 24 or at 48 hours of age. The kidneys from pups were excised 60-90 min later, fixed, and immunostained with antibodies to E-cadherin as described before [6]. After blocking in 3% BSA in PBS, and washing in 0.1 M sodium phosphate buffer pH 8, they were incubated in complete Click-iT reaction buffer for 90 min at room temperature, washed and imaged by confocal microscopy.

## Authors' contributions

EB, HL, SP, and LP designed the study; EB, HL, SP, and LP performed the studies; EB, DJ, SP, and LP analyzed the data; EB, SP, and LP. drafted the manuscript. All authors approved the content of the manuscript.

## Supplementary Material

Additional file 1 **Optical section of Tg(Crym-EGFP)82Gsat/Mmcd transgenic mouse kidney**. a) Strong GFP expression is seen in both the Cited1(+) (red) and Cited1(-) (arrows) capping mesenchyme at P2 in the Tg(Crym-EGFP)82Gsat/Mmcd mouse. GFP (green) is seen in the cap surrounding the branch tips of the ureteric bud and does not extend into the stroma. Ecadherin (blue); b) The abrupt change in character of the cap between P2 and P3 is accompanied by loss of Cited1 immunostaining. Click here for file

Additional file 2 **Optical section through transgenic mouse kidney at P0**. GFP is expressed at birth at lower intensity in the renal vesicles of the Tg(Crym-EGFP)82Gsat/Mmcd mouse than in the capping mesenchyme. The optical section through the nephrogenic region shows a portion of a renal vesicle (encircled), defined by morphological criteria of a central cavity in a deeper plane. Click here for file

Additional file 3 **Image of the FACS plot of cells collected for RNA measurements**. The GFP-positive cells with the highest level of expression were collected by FACS. Gating was set at a constant level as seen from birth to P3. After birth, both the fraction of GFP cells and the level of GFP intensity (peak shifts to the left) decreased. Click here for file

Additional file 4 **List of genes with changing levels of expression by microarray after birth**. Relative expression levels of 2000 genes with significant change in level of expression by ANOVA in the multi-potential progenitors after birth in mice. Levels of expression obtained in triplicate at birth (P0) and each 24-hour interval afterwards (P1-P4) were combined. The data is Log2 transformed. Click here for file

Additional file 5 **List of genes with a change in level of expression by RNA-Seq between P0 and P2**. RNA-Seq raw counts, normalized counts, fold change, and level of significance are shown for those genes with a significant difference in expression between P0 and P2. Click here for file

Additional file 6 **Functional sets of genes with a change in level by RNA-Seq between P0 and P2**. Genes identified by Partek Genomic Suites to significantly change were analyzed by ToppGene to identify functional classes. Click here for file

Additional file 7 **List of genes with changes in transcript levels between P1 and P4**. List of genes identified by GeneSpring analysis of microarrays. Click here for file

Additional file 8 **Functional sets of genes with transcript levels that decrease or increase between P1 and P4**. Genes identified by GeneSpring to significantly change were analyzed by ToppGene to identify functional classes. Click here for file
