# Supplementary figures and images for: Microarrays and RNA-Seq identify molecular mechanisms driving the end of nephron production
Source: BMC Dev Biol. 2011 Mar 12;11:15. doi: 10.1186/1471-213X-11-15 (PMC3065427; doi:10.1186/1471-213X-11-15)

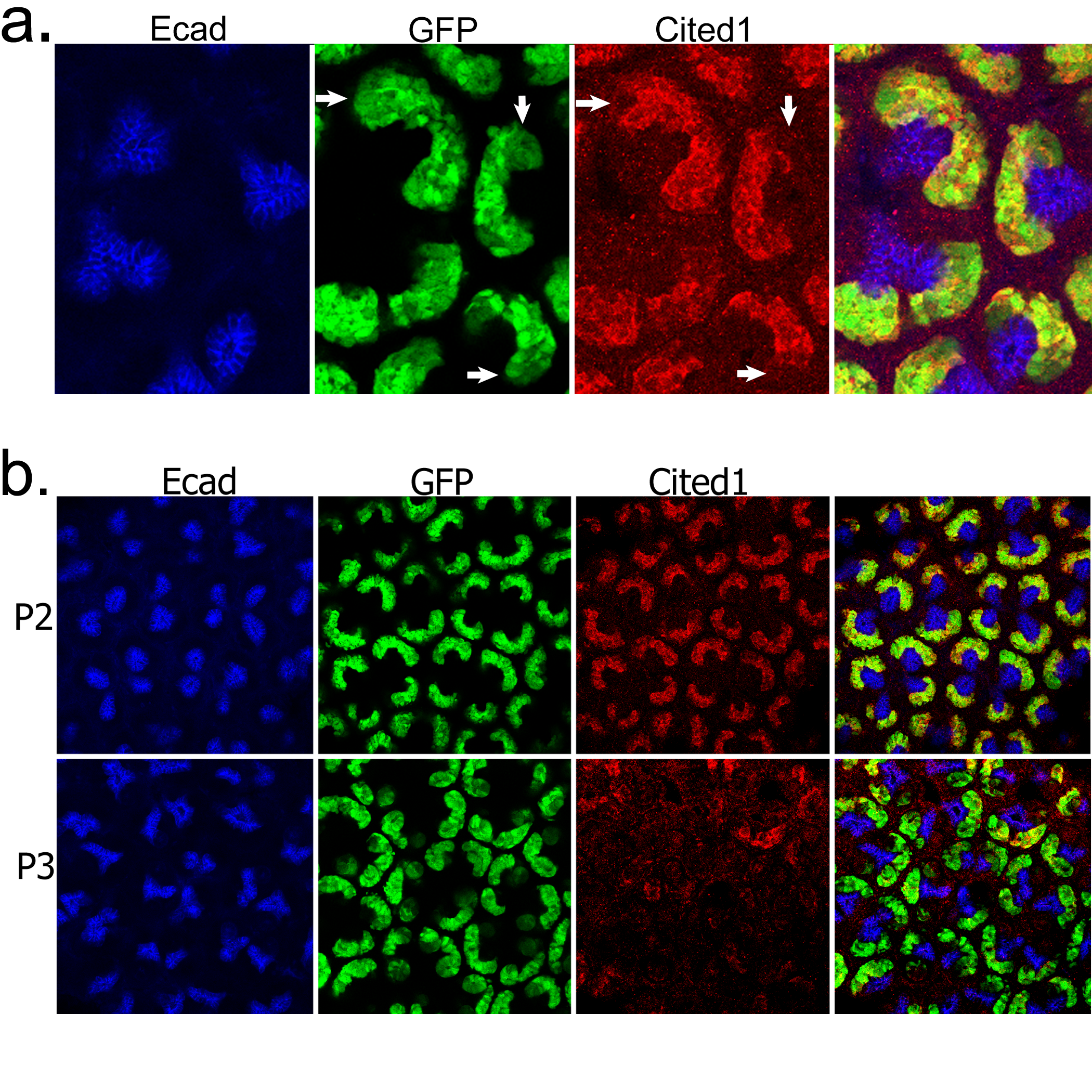

Supplement: Additional file 1 — Optical section of Tg(Crym-EGFP)82Gsat/Mmcd transgenic mouse kidney. a) Strong GFP expression is seen in both the Cited1(+) (red) and Cited1(-) (arrows) capping mesenchyme at P2 in the Tg(Crym-EGFP)82Gsat/Mmcd mouse. GFP (green) is seen in the cap surrounding the branch tips of the ureteric bud and does not extend into the stroma. Ecadherin (blue); b) The abrupt change in character of the cap between P2 and P3 is accompanied by loss of Cited1 immunostaining. [file 1471-213X-11-15-S1.TIFF]

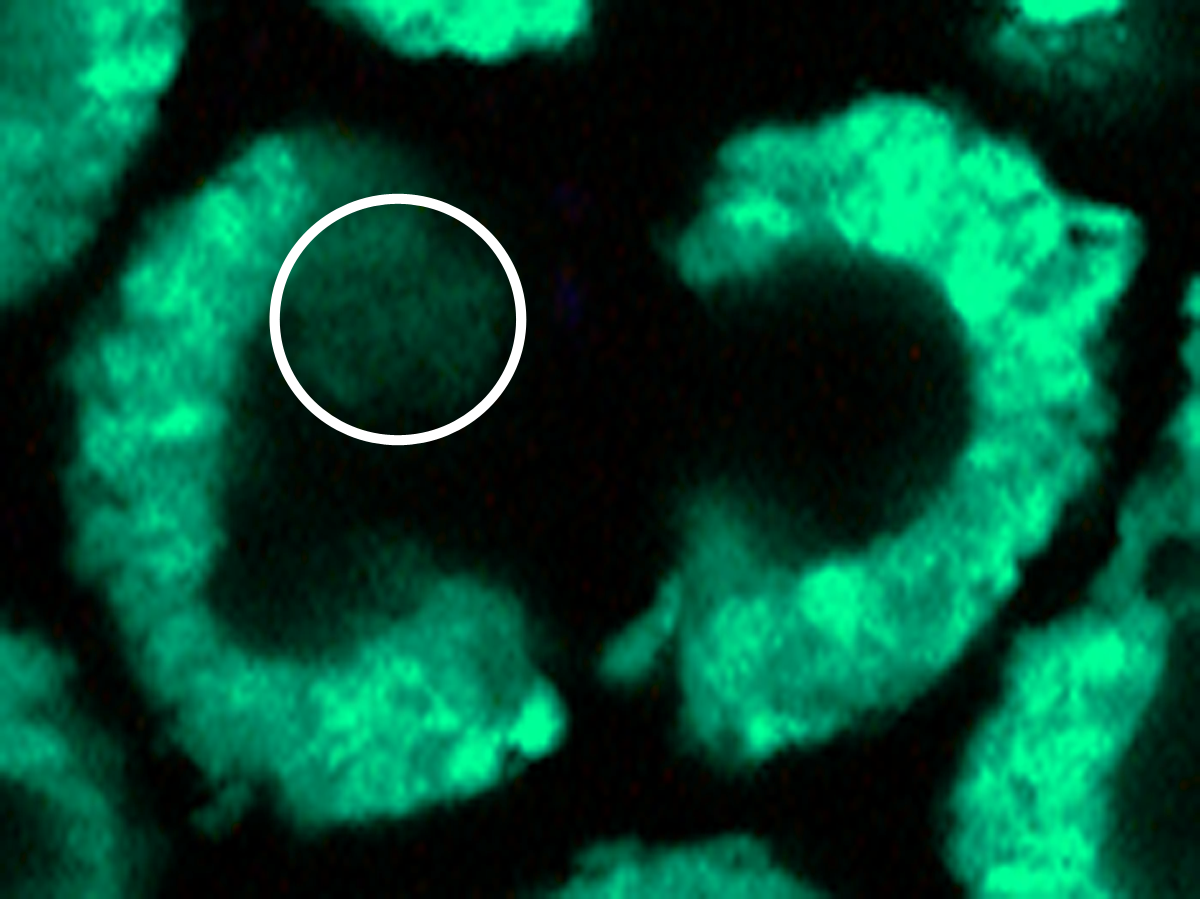

Supplement: Additional file 2 — Optical section through transgenic mouse kidney at P0. GFP is expressed at birth at lower intensity in the renal vesicles of the Tg(Crym-EGFP)82Gsat/Mmcd mouse than in the capping mesenchyme. The optical section through the nephrogenic region shows a portion of a renal vesicle (encircled), defined by morphological criteria of a central cavity in a deeper plane. [file 1471-213X-11-15-S2.TIFF]

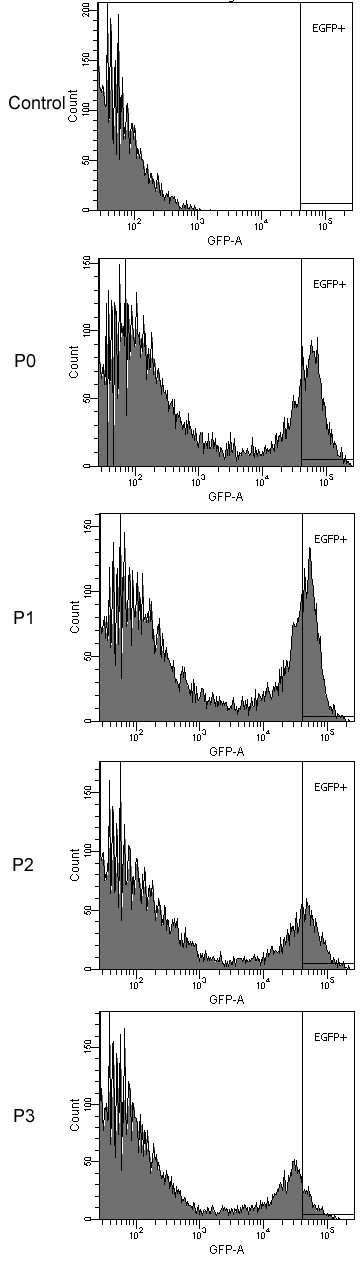

Supplement: Additional file 3 — Image of the FACS plot of cells collected for RNA measurements. The GFP-positive cells with the highest level of expression were collected by FACS. Gating was set at a constant level as seen from birth to P3. After birth, both the fraction of GFP cells and the level of GFP intensity (peak shifts to the left) decreased. [file 1471-213X-11-15-S3.TIFF]
